# Optimizing research publication of nurse researchers through a mentored writing retreat programme (MWRP): A participatory action research protocol

**DOI:** 10.1016/j.mex.2026.103943

**Published:** 2026-05-05

**Authors:** Remya U Rajendran, Mamatha Shivananda Pai, Baby S Nayak, Judith Angelitta Noronha, Vimala Ramoo, Srinivas Mutalik, Vasudeva Guddattu, Vishnu Renjith

**Affiliations:** a Department of Child Health Nursing, Manipal College of Nursing, Manipal Academy of Higher Education, Manipal, Udupi, Karnataka, India; b Department of Obstetrics&Gynaecological Nursing, Manipal College of Nursing, Manipal Academy of Higher Education, Manipal, Udupi, Karnataka, India; c Department of Nursing Science, Faculty of Medicine, University Malaya Medical Centre, Kuala Lumpur, Malaysia; d Manipal College of Pharmaceutical Sciences, Manipal Academy of Higher Education, Manipal, Udupi, Karnataka, India; e Department of Data Science, Prasanna School of Public Health, Manipal Academy of Higher Education, Manipal, Udupi, Karnataka, India; f School of Nursing & Midwifery, Royal College of Surgeons in Ireland, Dublin, Ireland. Honorary Senior Lecturer- School of Medicine, Cardiff University, United Kingdom

**Keywords:** Nurse researchers, Research publication, Mentored writing retreat programme, Participatory action research, Scientific writing

## Abstract

**Background:**

Nurse researchers often face challenges in publishing their work due to limited writing skills, inadequate mentorship, and insufficient institutional support. Strengthening research dissemination is essential for improving evidence-based practice, professional growth, and the visibility of nursing scholarship.

**Objective:**

Thisprotocol aims to identify barriers and facilitators to research publication among nurse researchers and to evaluate the effectiveness of a Mentored Writing Retreat Programme (MWRP) in optimizing the publication process.

**Methods:**

A participatory action research design will be adopted and implemented in two phases. **Phase I** will examine publication barriers and facilitators through a survey and focus group discussions among nurse researchers across Karnataka (*n* = 317). **Phase II** will use a quasi-experimental pre-test–post-test design with intervention and control groups (*n* = 50 per group). The MWRP will be developed based on Phase I findings and will include structured workshops, mentoring, and guided manuscript development over four months, followed by structured follow-up after six months post-intervention.

**Expected Outcomes:**

The protocol is expected to generate comprehensive evidence on challenges affecting research publication and to determine whether a structured writing retreat improves writing skills, increases publication readiness, and enhances scholarly productivity among nurse researchers. The findings will help inform future strategies to strengthen research capacity and enhance publication outcomes in nursing.

Protocol Outline:

## Abstract (author highlights)

• Identify publication barriers and facilitators among nurse researchers.
• Develop and deliver a structured Mentored Writing Retreat Programme.
• Evaluate changes in participants’ publication-related skills and outcomes.

## Graphical abstract

**Figure:**
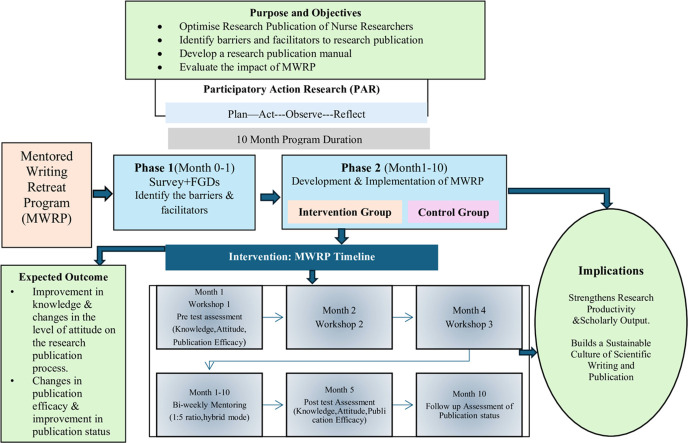

**Specifications table**

**Table:** 

Subject area	Medicine and Dentistry
**More specific subject area**	Scientific writing, research publication, and mentoring interventions for nurse researchers
**Name of your protocol**	Mentored Writing Retreat to Strengthen Research Publication Among Nurses
**Reagents/tools**	**Demographic Proforma** **DiaZePam Survey Questionnaire** Focus group Interview guide **Structured Knowledge Questionnaire** **Attitude Scale** **Publication Efficacy Scale** **Publication Status Checklist**
**Experimental design**	A two-phase participatory action research (PAR) design: **Phase I:** Survey and focus group discussions to identify barriers and facilitators to research publication among nurse researchers. **Phase II:** Quasi-experimental pretest–posttest design evaluating the impact of a Mentored Writing Retreat Programme (MWRP) delivered through three structured workshops over four months.
**Trial registration**	CTRI/2023/09/057470 [Registered on: 12/09/2023]
**Ethics**	This study was approved by the Institutional Ethics Committee of Kasturba Medical College(KMC), Manipal. **Human subjects statement:** Informed consent was obtained from all participants prior to survey participation, focus group discussions, and enrolment in the intervention programme. **Animal research:** Not applicable—this study does not involve animal subjects. **Social media data:** Not applicable—no social media data were used.
**Value of the Protocol**	• Provides a structured, replicable model (MWRP) to enhance writing skills, publication efficacy, and scholarly productivity among nurse researchers. • Uses participatory action research to co-develop a publication manual and tailor training based on real-world barriers and facilitators. • Supports capacity-building and strengthens research dissemination in nursing institutions, contributing to improved evidence-based practice.

## Background

Scientific evidence forms the basis for improving clinical decision-making and patient outcomes, and its dissemination through publication is essential for strengthening nursing practice[1,2]. Nursing research continuously generates new knowledge, while evidence-based practice ensures that this knowledge is appropriately translated into clinical care [3,4]. The ability to communicate research findings effectively through scientific writing is therefore crucial for expanding the evidence base within the nursing profession [5]. For this reason, training programmes that enhance research communication skills and support the translation of evidence into practice are increasingly emphasized in health sciences education[6].

Despite the growing need for scholarly output, nurses and other health professionals face significant challenges in preparing manuscripts and navigating the publication process. Several studies across health sciences have identified barriers such as limited writing skills, inadequate mentorship, lack of institutional support, low research confidence, and limited awareness of journal requirements [[7], [8], [9]]. Rejection of manuscripts due to poor scientific writing remains common, highlighting the importance of targeted writing support for researchers[10]. Structured scientific writing programmes including workshops, peer writing groups, and editorial mentoring have been shown to enhance writing proficiency and support manuscript development[[11], [12], [13]].

The academic context in India underscores an urgent need for initiatives that strengthen scientific writing capacity. Although research output is increasing, concerns persist regarding the quality of publications [14]. An analysis of research productivity in Indian institutions indicated substantial variability, with many colleges producing few or no publications over extended periods[15]. Challenges in manuscript writing, journal selection, and submission procedures have been consistently reported among researchers across disciplines[16]. Interventions such as writing workshops and coaching programmes have been recommended to address these gaps[17,18]. For nursing professionals in particular, structured writing support has been associated with improved writing confidence and manuscript completion[19].

Barriers to publication are multifaceted. External challenges include limited funding, insufficient research infrastructure, high publication charges, and prolonged review times [20,21]. Internal barriers include lack of time, inadequate writing skills, and difficulty with academic English[22,23]. The availability of mentorship, technical guidance, and feedback mechanisms has been identified as a key facilitator for overcoming these obstacles[20,24]. Previous studies also highlight that although many nursing faculty members show positive attitudes toward research, confidence in writing and publishing remains limited [25,26].

These contextual factors indicate the need for structured, practical, and accessible scientific writing support models for academic nurses. Short-term, focused writing programmes may offer an efficient approach for researchers who cannot participate in long-duration research courses[27]. This protocol is situated within this broader context. It outlines a structured approach to exploring publication challenges among nurse researchers and describes the development of a Mentored Writing Retreat Programme designed to address skill-based needs through a participatory, capacity-building process.

## Description of protocol

This protocol provides a reproducible, step-by-step method to optimise research publication among nurse researchers through the Mentored Writing Retreat Programme (MWRP) developed using Participatory Action Research (PAR). The protocol is designed to enable other researchers and academic institutions to replicate the intervention, data collection methods, and evaluation procedures.

### Aim of the study

The study aims to optimize research publication of Nurse Researchers through a Mentored Writing Retreat Programme (MWRP). We will address the following objectives,

1. Identify the barriers and facilitators to research publication as perceived by nurse researchers, measured by the Diazepam Survey questionnaire and focus group discussion.
2. Develop a research publication Manual.
3. Evaluate the impact of the Mentored Writing Retreat Programme (MWRP) on optimising the research publication process of nurse researchers in terms of,
• improvement in knowledge regarding research publication,
• changes in the level of attitude on the research publication process,
• changes in publication efficacy and
• improvement in the publication status.

### Study design

A participatory action research approach will be used throughout the study. Participatory action research (PAR) refers to a research method, typically concerned with organizational self-assessment, in which the subjects of the study"participate with the professional researcher throughout the research process from the initial design to the final presentation of the results and discussion of their action implications [28]

#### Design framework

**Fig. 1:**
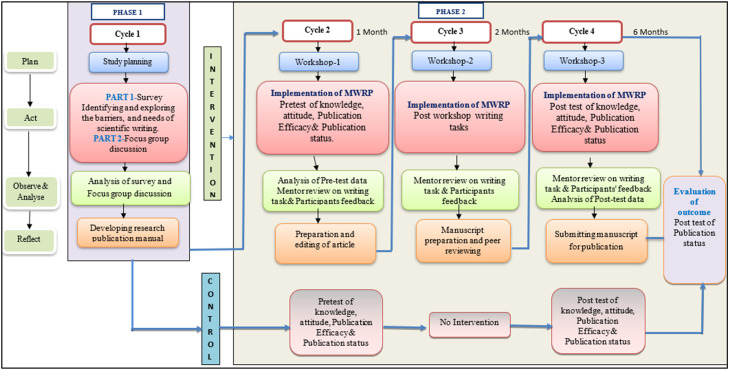
Framework of MWRP.

Participatory Action Research (PAR) involves four phases,1) plan, 2) act, 3) observe and 4) reflect [29]. A detailed study design framework using PAR in the implementation of MWRP is depicted in Fig. 1.

The development and implementation of the MWRP will follow four iterative PAR cycles:

• Plan: Identification of barriers and facilitators through surveys and focus group discussions.
• Act: Development and implementation of the MWRP and research publication manual.
• Observe: Monitoring participant progress, engagement, and outcomes during workshops and mentoring.
• Reflect: Evaluation of feedback from participants and mentors to refine workshop content, mentoring strategies, and delivery methods.

Each cycle will inform subsequent modifications, ensuring that the programme remains contextually relevant, responsive to participant needs, and continuously improved.

*Phase 1: Exploratory Survey on Barriers and Facilitators for Research Publication.*

Phase I of the study is structured as an explorative investigation aiming to identify the barriers and facilitators for research publication as perceived by nursing professionals employed in nursing institutions. This phase employs a combination of methods, including a descriptive survey and focus group discussions.

The primary objective of Phase I is to gain insights into the challenges and supportive factors encountered by nursing professionals in the process of publishing research findings. Through the descriptive survey, participants will provide valuable information regarding their experiences, perceptions, and the various obstacles hindering or facilitating their engagement in research publication activities.

Additionally, focus group discussions will be conducted to delve deeper into the identified themes and gather rich qualitative data. These discussions will offer a platform for participants to express their perspectives, share experiences, and offer suggestions for overcoming barriers to research publication.

The findings from Phase I will serve as a foundation for informing the development and implementation of interventions aimed at enhancing the research publication process among nurse researchers.

*Phase 2: Impact of Mentored Writing Retreat Programme (MWRP).*

In Phase II, the study shifts focus towards evaluating the impact of the Mentored Writing Retreat Programme (MWRP) on optimizing the research publication process among nurse researchers. This phase adopts a quasi-experimental design, specifically employing a Pretest-Posttest approach with both control and interventional groups.

Before the implementation of the MWRP, pre-test assessments will be administered to nurse researchers in both the interventional and control groups. These assessments will cover key domains including knowledge, attitude, publication efficacy, and publication status. This baseline assessment will provide a benchmark against which post-intervention outcomes can be compared.

Subsequently, the Writing Retreat Program will be developed based on the identified needs and requirements of nurse researchers, as revealed through a comprehensive need assessment survey. The intervention will be exclusively implemented for the nurse researchers in the interventional group through a series of three workshops, each spanning eight hours and covering relevant content areas, activities, learning materials, and feedback mechanisms.

Throughout the workshops, participants will be assigned writing tasks after each session, which will then be reviewed by the workshop facilitator. This iterative process aims to provide personalized feedback and guidance to participants, thereby enhancing their writing skills and proficiency. Following the completion of the workshops, post-testing will be conducted to assess changes in knowledge, attitude, publication efficacy, and publication status among both the control and intervention groups at four months following the conclusion of the final workshop session. The publication status will also be assessed after a six-month follow-up.

### Setting

The setting of the study will encompass nursing institutions affiliated with health sciences universities in Karnataka, India, where nursing faculty members holding MSc or PhD qualifications, as well as those currently pursuing PhDs, will be targeted for participation. With over 500 nursing colleges distributed across Karnataka, this setting offers a diverse landscape for exploring research publication experiences, challenges, and perceptions among nursing faculty. These institutions, affiliated with esteemed health sciences universities, serve as hubs for nursing education, research, and practice, contributing significantly to the healthcare workforce and academic development within the region. The presence of a large number of nursing colleges underscores the breadth and depth of the nursing education system in Karnataka, providing ample opportunities for sampling and data collection to gain insights into the research culture and scholarly activities of nursing faculty members.

### Study population

#### Phase 1

The study population for Phase 1 will be comprised of nurse researchers affiliated with nursing institutions offering postgraduate programs in Karnataka, India. These nurse researchers include faculty members with postgraduate qualifications and above, as well as PhD scholars actively engaged in research endeavors within the specified region.

Specifically, participants for Phase 1 will be drawn from individuals who have participated in the survey conducted as part of the study. Furthermore, the inclusion criteria will require that participants have conducted at least one research study and have reported encountering at least one barrier as identified in Part 1 of the survey. This will ensure that participants have direct experience with the challenges and barriers associated with research publication.

Exclusions from the Phase 1 study population will encompass nurse researchers who have previously received training on the research publication process and those who are unwilling to participate in the study. This exclusion criterion will ensure a focused and representative sample for investigating the research publication experiences and challenges among nurse researchers within the targeted setting, without potential confounding factors introduced by prior training or lack of willingness to engage in the study.

#### Phase 2

Nurse researchers who demonstrate a willingness to publish, receive mentorship, and prepare manuscripts will be chosen as participants for Phase 2. The inclusion criteria will require that participants have given their consent to participate in the study and meet the eligibility criteria established during Phase 1.

Phase 2 will include mentors who are willing to engage in mentoring activities related to research publication and manuscript preparation. Preference will be given to mentors who possess an h-index greater than 5, indicating a significant level of research productivity and expertise in the field. These mentors will play a crucial role in providing guidance and support to nurse researchers throughout the publication process.

Pre-test assessments will be conducted for both the interventional and control groups, encompassing nurse researchers who meet the predefined inclusion criteria. These assessments cover key domains including knowledge, attitude, publication efficacy, and publication status, providing baseline data for evaluating the impact of the Mentored Writing Retreat Programme (MWRP) intervention.

**Sample size.**

**Phase 1:** The sample size for survey was calculated using the single population proportion formula,

$$n=\frac{{\left(Z_{1-\frac{\alpha }{2}}\right)}^{2}pq}{{\left(d\right)}^{2}}$$

$Z_{1-\frac{\propto }{2}}$ = 1.96 at α = 0.05, *p* = Proportion, q = 1-p, d = allowable error (5% of p).

$$n=\frac{{\left(1.96\right)}^{2}x\;\;0.61\left(1-0.61\right)}{{\left(.03\right)}^{2}}=317$$

Focus group discussions (FGDs) will be conducted until data saturation is achieved.

**Phase 2:** The sample size was derived using the formula for two independent groups,

$$n=\frac{{\left(Z_{1-\frac{\alpha }{2}}+Z_{1-\beta }\right)}^{2}\sigma^{2}}{d^{2}},$$

$Z_{1-\frac{\propto }{2}}$ = 1.96 at α = 0.05, $Z_{1-\beta }$ = 0.84 at 80% power.

σ = Standard deviation difference of knowledge scores. d = Difference in pre- and post-intervention mean scores of knowledge.

The expected mean differences (d₁ = 2.26 and d₂ = 2.55) were derived from pilot observations conducted during preliminary implementation of similar training sessions.

To ensure a conservative and robust estimate, the average of the two values was considered for calculation:

$$d=\frac{2.26+2.55}{2}=2.40$$

The pooled standard deviation was calculated using: $\sigma =\sqrt{\frac{\sigma_{1}^{2}+\sigma_{2}^{2}}{2}}$where:

$$\sigma_{1}=20.7,\;\;\sigma_{2}=22.1\;\sigma =\sqrt{\frac{{\left(20.7\right)}^{2}+{\left(22.1\right)}^{2}}{2}}\approx 21.4$$

Substituting values into the formula:

$$n=\frac{{\left(1.96+0.84\right)}^{2}\times {\left(21.4\right)}^{2}}{{\left(2.40\right)}^{2}}$$

The calculated sample size was 46 participants per group (*n* = 46), and an attrition rate of 10%, the required sample size per group is approximately 50 participants.

A total of 100 nurse researchers (50 in each group) who met the inclusion criteria were included in the second phase of the study.

### Study variables

This study investigates the effects of a Mentored Writing Retreat Programme (MWRP) as the independent variable on several dependent variables. These dependent variables encompass Knowledge, Attitude, Publication Efficacy, and Publication Status. Through analysing these variables, the study aims to understand the impact of MWRP on participants' knowledge, attitudes, efficacy in publishing, and their actual publication status, offering insights into the effectiveness of such programs in enhancing scholarly productivity and publication success among researchers.

Operational Definitions

• Knowledge: Knowledge refers to the participants’ understanding of the research publication process, including manuscript preparation, journal selection, and submission procedures. Score range 0–30; higher scores indicate better knowledge.
• Attitude: Attitude refers to the participants’ perceptions, beliefs, and disposition towards research publication and engagement in scholarly writing activities.Score range 24–120; higher scores indicate positive attitude.
• Publication efficacy: Publication efficacy refers to the participants’ self-reported confidence in their ability to successfully prepare, submit, and publish a research manuscript in indexed journals.Score range 12–60; higher scores indicate higher confidence.
• Publication status: Number of manuscripts completed/submitted/published.

### Description of the tool

All tools used were either adapted from validated instruments or developed by the researcher following standard tool development guidelines. Tools underwent content validation by seven subject experts in nursing education, research, and biostatistics, and a pilot test was conducted among 30 nurse researchers not included in the main study. Reliability was established using Cronbach’s alpha for internal consistency (Table 1).

**Table 1:** Description of the research tools.

Phase	Name of the Tool	Type of the Tool	Number of Items	Prepared	Cronbach’s Alpha
Phase 1					
Tool 1	Demographic Proforma		15 items	Researcher	
Tool 2	DiaZePam survey questionnaire on barriers of nurse researchers to research publication[23]	Semi-structured Questionnaire	15 items with sub-items.	Developed by French researchers and available in the public domain.	0.786
Tool 3	Interview guide to explore the barriers and facilitators of nurse researchers on research publication	Semi-structured questions	10 open-ended questions	Researcher	
Phase 2					
Tool 4	Knowledge of the research publication process of nurse researchers	Structured knowledge questionnaire	30 multiple choice questions	Researcher	0.809
Tool 5	Attitude of nurse researchers toward research publication	5-point Likert scale	24 statements.	Researcher	0.795
Tool 6	Publication efficacy of nurse researchers	Rating scale	12 items	Researcher	0.856
Tool 7	Publication status of nurse researchers	Checklist	8 Items	Researcher	

The DiaZePam survey questionnaire [23], originally developed by French researchers, was used to assess barriers to publication. Although not standardized for Indian settings, it was content-validated and culturally adapted before use. Modified items were pretested, and reliability (Cronbach’s α = 0.786) was confirmed for the adapted version.

### Procedure for data collection

Phase I will begin with a quantitative survey to identify perceived barriers and facilitators among 317 nurse researchers across Karnataka. Following the survey, Focus Group Discussions (FGDs) will be conducted, each lasting 45–60 min with 10–12 participants, to gain deeper insights into thematic barriers and enablers.

The results from Phase I will guide the development of a Research Publication Manual and the MWRP intervention.

### Research publication manual

A structured Research Publication Manual will be developed and used as a core resource in the Mentored Writing Retreat Programme (MWRP). It will be informed by Phase I findings, literature, and expert input to ensure contextual relevance (Table 2).

**Table 2:** Description of mentored writing retreat programme (MWRP).

Components	Major area/topic to be covered	Time (Theory)	Time (Practical)	Learning resources	Writing task
Workshop 1 Basic components of scientific writing and publication	Introduction and course orientation 1. Components of a scientific paper • Structured style • Abstract & title • Figures & tables • Citations 2. Preparing an article for publication	4 h	4 h	Research publication manual Hands-on session: research article preparation	Preparing a research article
	Mentoring and follow-up				
Workshop 2 Steps in research publication process	Appraisal of a scholarly article Selection of journals Manuscript preparation & submission Review process Final approval	4 h	4 h	Manuscript samples Hands-on session: manuscript preparation	Manuscript preparation
	Mentoring and follow-up				
Workshop 3 Publication specific information	Editorial management systems Publication ethics & misconduct Editorial policies and processes	4 h	4 h	Interactive lectures Resources for publication	Manuscript submission
	Mentoring and follow-up				

The manual will provide step-by-step guidance based on the IMRaD structure, covering development of the introduction, description of methods, presentation of results, and interpretation in discussion. It will also include sections on journal selection (scope, indexing, and avoiding predatory journals), publication ethics (authorship, plagiarism, and research integrity), and the manuscript submission process (cover letter preparation, submission systems, and responding to peer review).

Additionally, the manual will include practical tools such as checklists, templates, and sample formats to support manuscript development. It will be used during workshops and mentoring sessions.

In Phase II, eligible participants (*n* = 100) will be randomly assigned to either an intervention (*n* = 50) or a control group (*n* = 50). The intervention group will participate in the MWRP, comprising three workshops conducted over a four-month period. The control group will receive no intervention during this period. Pre- and post-assessments will be conducted for both groups at baseline, as well as at four- and six-month post-intervention.

All data will be coded anonymously, stored securely, and accessible only to the principal investigator.

Recruitment and Allocation Procedures.

Participants for Phase I will be recruited from nursing institutions across Karnataka through official communication with institutional heads and dissemination of study information via email and professional networks. Eligible nurse researchers who meet the inclusion criteria and provide informed consent will be enrolled in the survey. Participants for focus group discussions will be purposively selected from survey respondents to ensure diversity of experiences and perspectives.

For Phase II, participants will be recruited from Phase I respondents who indicate willingness to participate in the Mentored Writing Retreat Programme (MWRP) and meet the predefined eligibility criteria. After obtaining informed consent, eligible participants will be enrolled and assigned unique identification codes.

Given the quasi-experimental design, participants will be allocated into intervention and control groups using a non-randomized approach. Allocation will be based on feasibility and participant availability, ensuring comparable group sizes (*n* = 50 per group). Efforts will be made to maintain baseline comparability between groups by considering key characteristics such as academic qualification, prior publication experience, and institutional affiliation.

The intervention group will receive the MWRP, while the control group will continue with routine academic activities without structured intervention during the study period. To ensure ethical fairness, the control group will be provided access to the training materials after completion of the study.

### Description of the intervention-mentored writing retreat programme (MWRP)

MWRP is a learning concept developed by the researcher to optimize the research publication of nurse researchers based on a participatory action research approach. Participatory action research emphasizes the involvement of participants throughout the study process.

The MWRP combines workshops, mentorship support, self-learning resources, interactive learning, and follow-up to support participants in preparing and submitting manuscripts to indexed journals.

Three workshop sessions will be conducted over a four-month period at various intervals, with follow-up to be conducted after six months. So, the total duration of the programme will be ten months. Participants will engage in comprehensive sessions covering the basic components of scientific writing and editing, including lectures, presentations, hands-on sessions, and feedback. Topics include Introduction and course orientation, Components of a scientific paper structure & style, Abstract & title, Figures & tables, Citations, and Preparing an article for publication. Additionally, steps in the research publication process will be explored, involving lectures, presentations, hands-on sessions, and feedback to develop skills in scientific article appraisal, journal selection, manuscript preparation & submission, the review process, and final manuscript approval. Furthermore, publication-specific information will be provided through interactive lectures, presentations, and feedback sessions, covering various terminologies such as peer review, journal indexing, impact indicators, editorial management systems, publication ethics & misconduct, and editorial policies and processes.

### Programme structure and timeline

The MWRP will be implemented over a 10-month period, comprising three workshops conducted over four months, followed by structured mentoring and follow-up:

• Month 1 (Week 1): Workshop 1 (8 h) - Introduction to scientific writing, manuscript structure, title and abstract development.
• Month 2 (Week 5): Workshop 2 (8 h) - Writing the introduction and methods, literature synthesis.
• Month 4 (Week 12): Workshop 3 (8 h) - Results, discussion, journal selection, and manuscript submission.
• Months 1–10: Bi-weekly mentoring sessions.
• Month 5: Post-test assessment (knowledge, attitude, efficacy).
• Month 10: Follow-up assessment (publication status).

Rationale: The spacing between workshops allows participants sufficient time to complete writing tasks, receive feedback, and iteratively improve their manuscripts.

### Mentoring structure and follow-up

A structured mentoring framework will be integrated throughout the programme:

• Mentor–mentee ratio: 1:5.
• Mode of mentoring: Hybrid (in-person during workshops and virtual follow-up via email or online platforms such as Microsoft Teams).
• Frequency: Bi-weekly sessions post workshops.
• Duration: 30–60 min per session.

Mentor responsibilities include:

• Providing individualized feedback on writing tasks.
• Guiding journal selection and submission processes
• Supporting manuscript revision following peer-review feedback.

Mentee responsibilities include:

• Timely completion of assigned writing tasks.
• Revising drafts based on mentor feedback.
• Active participation in mentoring sessions.

### Writing tasks and learning process

Each workshop will include structured writing assignments aligned with learning objectives:

• Workshop 1: Drafting title, abstract, and manuscript outline.
• Workshop 2: Writing introduction and methods sections.
• Workshop 3: Completing results, discussion, and submission-ready manuscript.

Feedback Mechanism:

• Written feedback will be taken from the participants after each workshop.

### Content coverage

The programme will include interactive sessions involving lectures, hands-on activities, and feedback, covering:

• Scientific paper structure and writing style.
• Abstract and title development.
• Figures, tables, and referencing.
• Manuscript preparation and submission.
• Journal selection and editorial processes.
• Peer review and revision strategies.
• Publication ethics and misconduct.
• Indexing, impact indicators, and editorial systems.

## Data analysis

Quantitative data will be analyzed using Jamovi 2.4.1 version. Descriptive statistics (mean, SD, frequency, and percentage) will summarize demographic data and baseline characteristics.

Inferential statistics:

• Paired *t*-test will compare pre- and post-test scores within groups.
• Repeated measures ANOVA will compare mean differences between groups, controlling for baseline values.
• The Cochran Q test will analyse categorical variables such as publication status.
• Significance will be set at *p* < 0.05.

Qualitative data from FGDs will undergo thematic analysis following Braun and Clarke’s (2006) six-step approach: familiarisation, coding, theme development, reviewing, defining, and reporting. The integration of quantitative and qualitative findings will be performed to provide a comprehensive understanding of the barriers, facilitators, and impact of the intervention.

Furthermore, the effectiveness of the workshop-based intervention will be evaluated by comparing means of knowledge, attitude, publication efficacy, and publication status before and after the workshop using *t*-tests, allowing for an assessment of any significant changes resulting from the intervention. This comprehensive data analysis approach aims to provide a thorough understanding of the factors influencing research publication among nurse researchers and the impact of the intervention on key outcomes.

### Ethical considerations

Ethical approval for this study has been obtained from the Institutional Ethics Committee. Administrative permissions will be secured from all participating institutions. Participants will receive a Participant Information Sheet (PIS) that details the study objectives, voluntary participation, and their right to withdraw without penalty.

Written informed consent will be obtained prior to participation. Confidentiality will be maintained through anonymized data storage, password-protected files, and restricted access. Data will be used solely for research purposes. No physical, psychological, or professional risks are anticipated. Findings will be disseminated through academic conferences and peer-reviewed journals, maintaining participant anonymity.

### Protocol validation

The need to strengthen scientific writing and publication capacity among nurse researchers has become increasingly evident, particularly in resource-constrained academic environments where structured mentorship and institutional support are limited. Global literature identifies persistent barriers such as inadequate research culture, limited writing confidence, lack of experienced mentors, and difficulty navigating the publication process all of which contribute to reduced scholarly productivity among nurses [17,[30], [31], [32]]. Evidence from international and national studies demonstrates that structured interventions such as writing retreats, mentored workshops, and peer-supported learning significantly improve writing self-efficacy, manuscript quality, and publication outcomes [[33], [34], [35]]. However, there is a notable gap in well-designed, context-specific mentorship programmes tailored for nursing researchers in India, particularly those grounded in participatory, action-oriented frameworks.

The Mentored Writing Retreat Programme (MWRP) was developed using a participatory action research (PAR) approach, enabling iterative refinement of the intervention through exploratory surveys, focus group discussions, and expert inputs. PAR-based programme development ensures contextual relevance, stakeholder ownership, and continuous adaptation features, which have been shown to increase the effectiveness and sustainability of educational interventions [36]. The programme integrates evidence-based elements including action-based mentoring, peer collaboration, hands-on manuscript development, and structured follow-up mechanisms, aligning with best practices in scientific writing pedagogy [37,38].

To ensure methodological rigour, all components of the protocol, including the research publication manual, training modules, assessment tools, and retreat structure, will undergo content validation by experts in scientific writing, nursing education, and research methodology. Expert validation processes willbe recommended to ensure clarity, relevance, and appropriateness of intervention materials. Preliminary pilot data obtained from three retreat cycles demonstrated high participant acceptability, active engagement, and successful achievement of session-wise manuscript development tasks. Early descriptive findings from pre- and post-assessment data will also indicate improvements in participants’ scientific writing confidence, understanding of the publication process, and progression of manuscript drafts, consistent with trends reported in similar writing intervention research [12,39].

While full results and discussion are not included in protocol manuscripts, the preliminary data support the feasibility, acceptability, and relevance of the MWRP. Follow-up mentor–mentee interactions further validated the practical usefulness of the manual and training activities, reinforcing the suitability of implementing the protocol on a larger scale. To the best of our knowledge, this protocol is novel and has not been previously implemented in nursing research settings in India. Comprehensive outcome data will be generated following the completion of Phase II of the study.

### Limitations

• The quasi-experimental design may not fully eliminate external factors influencing changes in publication-related outcomes, which could affect the internal validity of the protocol.
• The protocol relies on self-reported measures of knowledge, attitudes, and publication efficacy, which may be subject to social desirability or recall bias.
• The protocol is designed for nursing institutions in South India; therefore, its applicability to other regions or disciplines may be limited.
• Participant engagement and completion of manuscript tasks may vary based on workload, motivation, and competing academic responsibilities, potentially affecting the overall effectiveness of the intervention.

## Related research article

Challenges and needs of nurse researchers on research publication in health universities of Karnataka, India: a cross-sectional survey.

## For a published article

Challenges and needs of nurse researchers on research publication in health universities of Karnataka, India: a cross-sectional survey

## CRediT author statement

**Remya U Rajendran:** Conceptualization, Methodology, Resources, Writing – original draft, Writing – review & editing. **Mamatha Shivananda Pai**: Conceptualization, Funding acquisition, Methodology, Writing – review & editing. **Baby S Nayak:** Conceptualization, Methodology, Writing – review & editing. **Judith Angelitta Noronha**: Conceptualization, Writing – review & editing. **Vimala Ramoo:** Writing – review & editing, **Srinivas Mutalik:** Writing – review & editing, **Vasudeva Guddattu:** Writing – review & editing, **Vishnu Renjith:** Methodology, Resources, Writing – review & editing.

## Declaration of competing interest

The authors declare that they have no known competing financial interests or personal relationships that could have appeared to influence the work reported in this paper.

## Data Availability

No data was used for the research described in the article.
